# Chemical Composition, Antioxidant, Anticancer, and Antibacterial Activities of Roots and Seeds of *Ammi visnaga* L. Methanol Extract

**DOI:** 10.3390/ph17010121

**Published:** 2024-01-17

**Authors:** Ibrahim M. Aziz, Rawan M. Alshalan, Humaira Rizwana, Fetoon Alkhelaiwi, Abdulaziz M. Almuqrin, Reem M. Aljowaie, Noorah A. Alkubaisi

**Affiliations:** 1 Department of Botany and Microbiology, College of Science, King Saud University, Riyadh 11451, Saudi Arabia; ralshalaan@ksu.edu.sa (R.M.A.); hrizwana@ksu.edu.sa (H.R.); falklewiy@ksu.edu.sa (F.A.); raljowaie@ksu.edu.sa (R.M.A.); nalkubaisi@ksu.edu.sa (N.A.A.); 2 Department of Clinical Laboratory Sciences, College of Applied Medical Sciences, King Saud University, Riyadh 12372, Saudi Arabia; aalmuqrin@ksu.edu.sa

**Keywords:** natural products, medicinal plants, anticancer potential, herbal treatment, lung disease

## Abstract

For centuries, plants and their components have been harnessed for therapeutic purposes, with *Ammi visnaga* L. (Khella) being no exception to this rich tradition. While existing studies have shed light on the cytotoxic and antimicrobial properties of seed extracts, there remains a noticeable gap in research about the antimicrobial, antioxidant, and anticancer potential of root extracts. This study seeks to address this gap by systematically examining methanol extracts derived from the roots of *A. visnaga* L. and comparing their effects with those of seed extracts specifically against breast cancer cells. Notably, absent from previous investigations, this study focuses on the comparative analysis of the antimicrobial, antioxidant, and anticancer activities of both root and seed extracts. The methanol extract obtained from *A. visnaga* L. seeds demonstrated a notably higher level of total phenolic content (TPC) than its root counterpart, measuring 366.57 ± 2.86 and 270.78 ± 2.86 mg GAE/g dry weight of the dry extract, respectively. In the evaluation of antioxidant activities using the DPPH method, the IC_50_ values for root and seed extracts were determined to be 193.46 ± 17.13 μg/mL and 227.19 ± 1.48 μg/mL, respectively. Turning our attention to cytotoxicity against breast cancer cells (MCF-7 and MDA-MB-231), both root and seed extracts displayed similar cytotoxic activities, with IC_50_ values of 92.45 ± 2.14 μg/mL and 75.43 ± 2.32 μg/mL, respectively. Furthermore, both root and seed extracts exhibited a noteworthy modulation of gene expression, upregulating the expression of caspase and Bax mRNA levels while concurrently suppressing the expression of anti-apoptotic genes (Bcl-xL and Bcl-2), thereby reinforcing their potential as anticancer agents. *A. visnaga* L. seed extract outperforms the root extract in antimicrobial activities, exhibiting lower minimum inhibitory concentrations (MICs) of 3.81 ± 0.24 to 125 ± 7.63 μg/mL. This highlights the seeds’ potential as potent antibacterial agents, expanding their role in disease prevention. Overall, this study underscores the diverse therapeutic potentials of *A. visnaga* L. roots and seeds, contributing to the understanding of plant-derived extracts in mitigating disease risks.

## 1. Introduction

Medicinal plant-based compounds provide an extensive array of chemical diversity, which plays a vital role in the exploration and advancement of contemporary medicinal therapies [1,2,3]. Over the past few decades, numerous natural plant-derived compounds have been identified, exhibiting antioxidative, anticancer, anti-inflammatory, and antibacterial properties explained by their ability to activate a variety of signaling transduction pathways [4,5,6,7,8]. Approximately 25% of current medicines are derived from plants, underscoring the profound impact of botanical sources in pharmaceuticals. Moreover, globally, up to 200 species are recognized as therapeutic plants, emphasizing the rich diversity and potential within the plant kingdom for medicinal applications [9].

*Ammi visnaga*, commonly known as Khella and Bishop’s Weed, is an annual aromatic herb belonging to the *Apiaceae* family. Originating from the Mediterranean region, this herb has proliferated globally, extending its presence to Europe, Asia, and North America. The widespread distribution of *A. visnaga* underscores its adaptability and its integration into diverse ecosystems beyond its native habitat [10,11]. For centuries, the utilization of plants and plant components in the treatment of diseases has been a pervasive practice, and *A. visnaga* L. stands as no exception to this historical trend [12]. Moreover, *A. visnaga* L. has earned recognition in the literature and works of diverse Arab chemists and physicians, attesting to its well-documented medicinal and therapeutic characteristics [12,13]. The therapeutic efficacy of *A. visnaga* L. is intricately linked to its rich phytochemical composition, encompassing diverse bioactive constituents such as furanochromones and pyranocoumarins. Recent years have witnessed a discernible surge in scholarly interest directed toward elucidating the pharmacological properties inherent to *A. visnaga* L. [14,15,16,17]. Furthermore, notable chemical constituents found in substantial concentrations in this botanical specimen encompass butanoic acid, 2-methyl-, pentyl ester, (Z)-β-ocimene, D-limonene, linalool, pulegone, lavandulyl-butyrate, marmesin, isoimperatorin, heraclenin, isopimpinellin, nonhydroxylic coumarins, ammirin, alloimperatorin, khellin, visnagin, and acetylated flavonoids, in addition to the essential oil [18,19]. The essential oil derived from *A. visnaga* L. has been demonstrated to possess notable antiviral, antibacterial, and larvicidal properties. Furthermore, the antioxidant activity of the plant can be attributed to its flavonoid content. These beneficial properties of *A. visnaga* have been recognized and utilized for therapeutic purposes for over 7000 years [12,20,21]. The odorant volatile compounds responsible for the aroma of *A. visnaga* are found in flowers, leaves, stems, peels, and roots [22]. Aqueous extracts of *A. visnaga* seeds have shown significant improvement in conditions such as uremia, nephrolithiasis, and hyperbilirubinemia. These beneficial effects have been reported in various studies [23,24,25]. In addition, it has been observed that the aqueous extract of *A. visnaga* exhibits potential benefits in reducing blood sugar levels and is believed to possess hypoglycemic effects in rats [26]. Previous research has provided evidence of the significant inhibitory effects of *A. visnaga* L. extracts on the growth of bacteria and fungi [27,28].

*A. visnaga* L. extracts have been reported to possess therapeutic potential in the treatment of stomach pains and moderate anginal symptoms. It is also used as a supportive therapy for minor respiratory tract obstruction in asthma or spastic bronchitis, as well as postoperative treatment for disorders related to the presence of urinary calculi. The plant and its extracts are also utilized as a lithotriptic drug in the treatment of kidney stones. It is also used internally as an emmenagogue to control menstruation, as a diuretic, and to treat vertigo and diabetes. Aerial parts infusions have also been used to cure headaches [12,29,30]. Additionally, these extracts have demonstrated the ability to reduce blood pressure and to alleviate bronchial spasms [31,32]. A recent study has provided evidence of the cytotoxic effects of *A. visnaga* seed extract on a human liver cancer cell line (HuH-7) [33].

Cytotoxic agents cause apoptosis in susceptible target cells by activating death signaling pathways. Chemotherapeutic agents induce apoptosis by activating death receptor systems simultaneously or sequentially, disrupting mitochondrial function and proteolytic processing of caspases [4]. Thus, the cell death pathway may occur in several sites; however, the precise molecular pathways for each medication and unique target cell have not been completely elucidated. In the present study, changes in caspase-3, -8, -9, and Bax (pro-apoptotic) genes and anti-apoptotic genes (Bcl-xL and Bcl-2) following apoptosis stimulation by methanol extracts of *A. visnaga* L. in MCF-7 and MDA-MB-231 cells was revealed. Apoptosis, or programmed cell death, is a critical component of many processes that occur in response to environmental stimuli. According to research, there are two primary apoptotic routes in mammalian cells: the extrinsic or death receptor pathway and the intrinsic or mitochondrial system. The activation of caspases is connected with the induction of cell death in both extrinsic and intrinsic apoptotic pathways [34]. Aspase-8 is mostly active in the extrinsic apoptotic route, while caspase-9 activation is associated with the mitochondrial or intrinsic pathway [35]. Caspases may be activated in anticancer treatment by stimulating the extrinsic route or by activating the intrinsic pathway at the mitochondria [36]. Increased Bax expression in breast cancer cells enhances sensitivity to apoptotic stimuli and reduces tumor growth [37]. Another anti-apoptotic protein that inhibits apoptosis is Bcl-xL [38]. Bcl-xL expression has been correlated to the progression of breast cancer [39]. While Bcl-xL suppresses apoptosis, it is regarded as a critical molecule in the induction of chemoresistance [40]. On the other hand, past research has extensively explored the cytotoxic and antibacterial effects of seed extracts; a comparative analysis with root extracts regarding antimicrobial, antioxidant, and anticancer properties remains unexplored. The chemical mechanisms underlying the biological effects of *A. visnaga* roots and seed extracts remain enigmatic. This study aims to fill this gap by evaluating the phytochemical composition and antioxidant properties of *A. visnaga* L. root extracts compared to seed extracts, utilizing the 1,1-diphenyl-2-picryl hydrazyl (DPPH) scavenging assay and 2,2′-azino-bis (3-ethylbenzothiazoline-6-sulfonic acid) (ABTS) assay. Additionally, it seeks to assess the potential effects of *A. visnaga* L. roots compared to seed extracts against breast cancer (MCF-7) and MDA-MB-231 cells. This study further aims to elucidate the underlying mechanisms through qPCR-based mRNA expression profiling of selected pro- and anti-apoptosis marker genes. Moreover, antibacterial activity against Gram-positive and Gram-negative bacteria will be explored using the minimum inhibitory concentration (MIC) and minimum bactericidal concentration (MBC) methods.

## 2. Results

### 2.1. Chemical Composition of Methanol Roots and Seeds Extracts of A. visnaga L.

**Figure 1:**
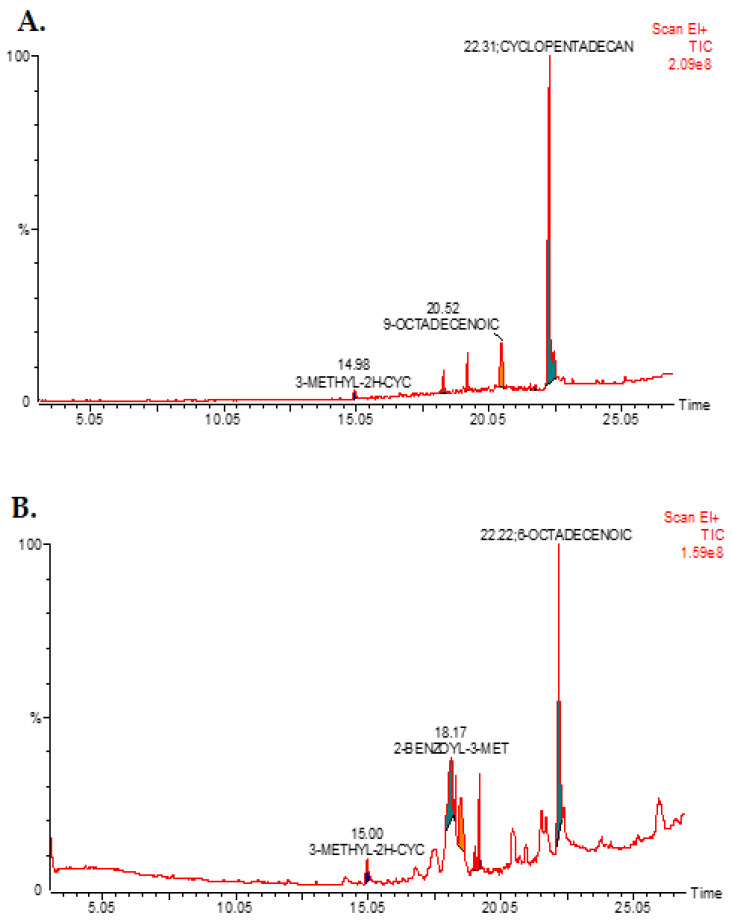
The GC-MS spectrum of the compounds extracted from *A. visnaga* L., specifically from the roots (**A**) and seeds (**B**), is shown in Figure 1A and Figure 1B, respectively. For the analysis, a methanol extract was used, and the GC-MS instrument was programmed for 30 min. Each peak observed in the spectrum represents an identified compound, and the presence of a large peak indicates the major compound in the extract.

GC-MS/FID analysis was conducted to analyze the methanol extract of *A. visnaga* L. roots and seeds for phytochemical components. The identified compounds are presented in Table 1 and Figure 1A. In the root methanol extract, the predominant phytochemical compound identified was Docosanolide (73.39%), followed by Angecin (10.75%) and 2,5-Dimethyl-5-Nitrohexanal (10.70%). On the other hand, the major phytochemical compound in the methanol extract of the seeds was 2-Benzoyl-3-Methyl-2,3-Diaza (54.60%), followed by 2-Methoxy-4-Propyl-Phenol (24.96%) and 6-Octadecenoic Acid (10.68%) (Table 2 and Figure 1B).

### 2.2. Antioxidant Activity

#### 2.2.1. Total Phenolic Content (TPC) and Total Flavonoid Content (TFC)

The methanol extract derived from the seeds of *A. visnaga* L. displayed a higher TPC level, measuring 366.57 ± 2.86 ± 4.09 mg GAE/g dry weight of the dry extract than the roots extract measured value of 270.78 ± 2.86 mg GAE/g dry weight of the dry extract, according to the correlation coefficient (R^2^ = 0.977). Regarding the TFC, the seeds of *A. visnaga* L. contained an amount of 242.10 ± 3.07 mg QE/g dry weight of the dry extract, while the roots exhibited a slightly lower TFC value of 214.46 ± 2.86 mg QE/g dry weight of the dry extract, according to the correlation coefficient (R^2^ = 0.965), indicating a highly significant correlation between the TFC and the extract from both roots and seeds of *A. visnaga* L.

#### 2.2.2. DPPH and ABTS Radical Scavenging Activity

**Figure 2:**
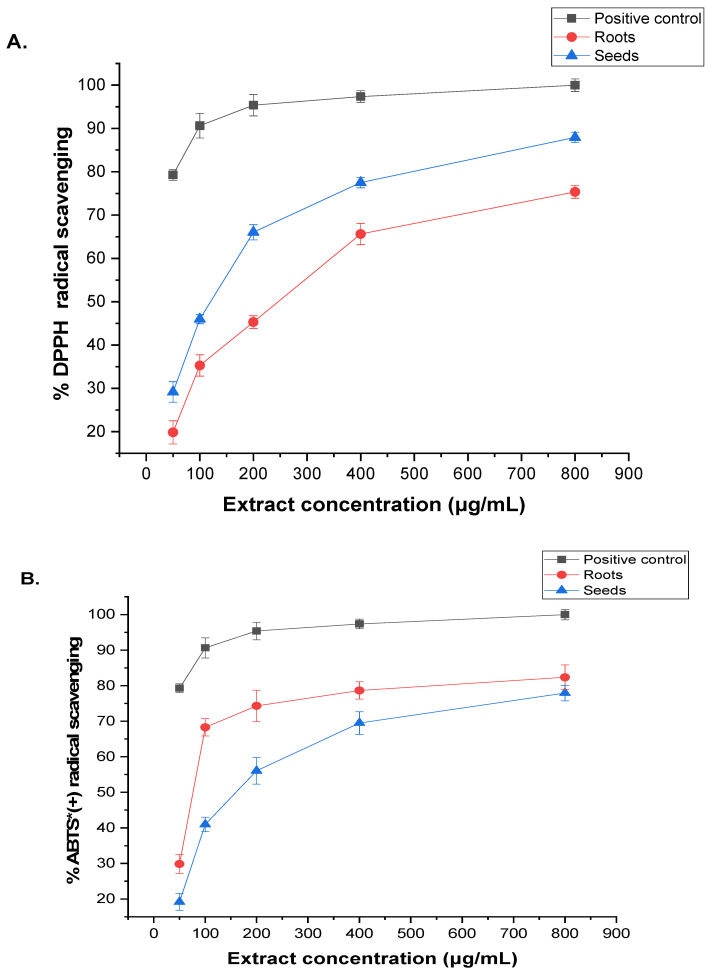
DPPH and ABTS estimation of (**A**) roots and (**B**) seeds of *A. visnaga* L. extracts. The presented statistics are mean values based on three replicates ± SD. * = radical cation.

The antioxidant capacity of the phytochemicals extracted from the roots and seeds of *A. visnaga* L. was assessed using two methods: DPPH and ABTS radical scavenging activity. The results, presented in Figure 2, were compared with vitamin C (IC_50_ of 83.45 ± 1.24 μg/mL). Higher concentrations of the extract led to enhanced DPPH and ABTS radical activities. In the DPPH assay, the root extract exhibited an IC50 value of 193.46 ± 17.13, while the ABTS assay showed a value of 188.74 ± 2.69 μg/mL (Figure 2A). Similarly, the seed extract demonstrated improved DPPH and ABTS radical scavenging abilities with increasing concentration, yielding IC_50_ values of 227.19 ± 1.48 and 221.94 ± 1.32 μg/mL, respectively (Figure 2B).

### 2.3. Cytotoxic Activity

**Figure 3:**
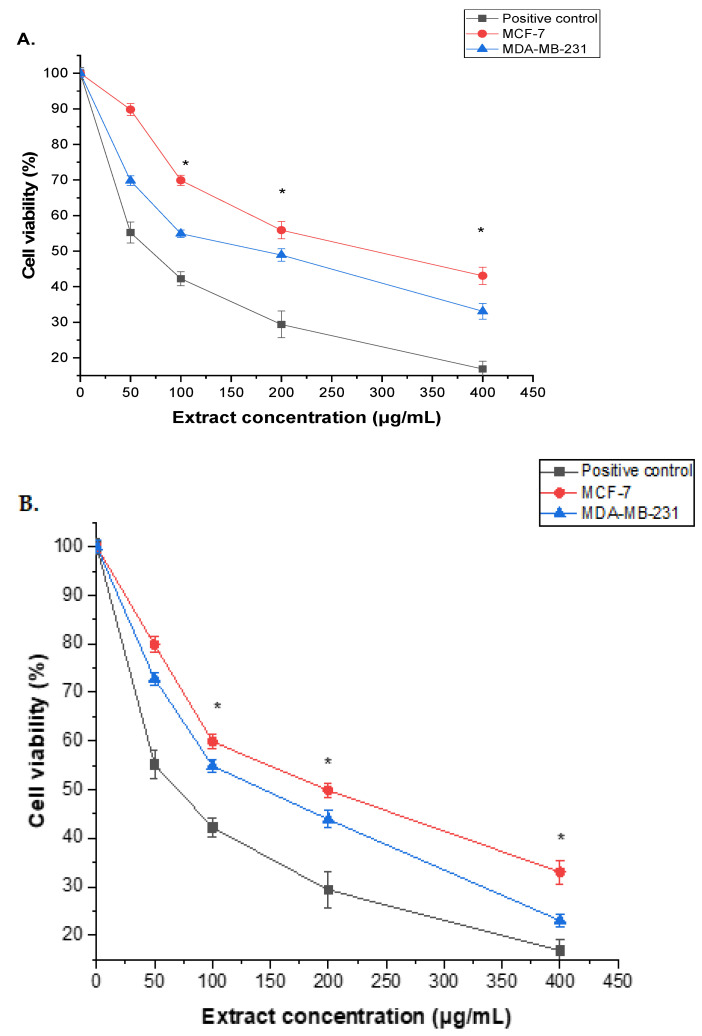
The cytotoxicity of *A. visnaga* L. extracts derived from roots (**A**) and seeds (**B**) was examined using the MTT assay for 24 h, with concentrations ranging from 0 to 400 μg/mL. The results, represented as the mean ± SD of three independent experiments, demonstrated a statistically significant difference from the control (* = *p* < 0.05). In particular, the cytotoxic effects of the extracts on MCF-7 and MDA-MB-231 cells were assessed.

Root and seed extracts of *A. visnaga* L. exhibited a dose-dependent inhibition of proliferation, as illustrated in (Figure 3). Remarkably, compared to the positive control (doxorubicin), the methanol extracts from both *A. visnaga* L. roots and seeds demonstrated robust cytotoxic activity against MCF-7 and MDA-MB-231 cells. The results indicated a significant (*p* < 0.05) difference between untreated and treated cells at varied doses. While 50 μg/mL of roots and seeds extracts showed limited inhibition of MCF-7 and MDA-MB-231 cell growth, higher concentrations of roots extract exhibited a gradient cytotoxic effect, with significant viability reduction (*p* < 0.05) at 100–400 μg/mL. The IC_50_ values of MCF-7 and MDA-MB-231 cells were 92.45 ± 2.14 and 75.43 ± 2.32 μg/mL, respectively (Figure 3A). Similar trends were observed with the seed extract, displaying IC_50_ values of 87.35 ± 2.44 and 72.49 ± 1.36 μg/mL for MCF-7 and MDA-MB-231 cells, respectively (Figure 3B). Notably, the seed extract demonstrated a more potent cytotoxic effect than the root extract against both cell lines.

### 2.4. Effects of A. visnaga L., Root and Seeds Extracts on MCF-7 and MDA-MB-231 Induced Apoptosis Signaling

**Figure 4:**
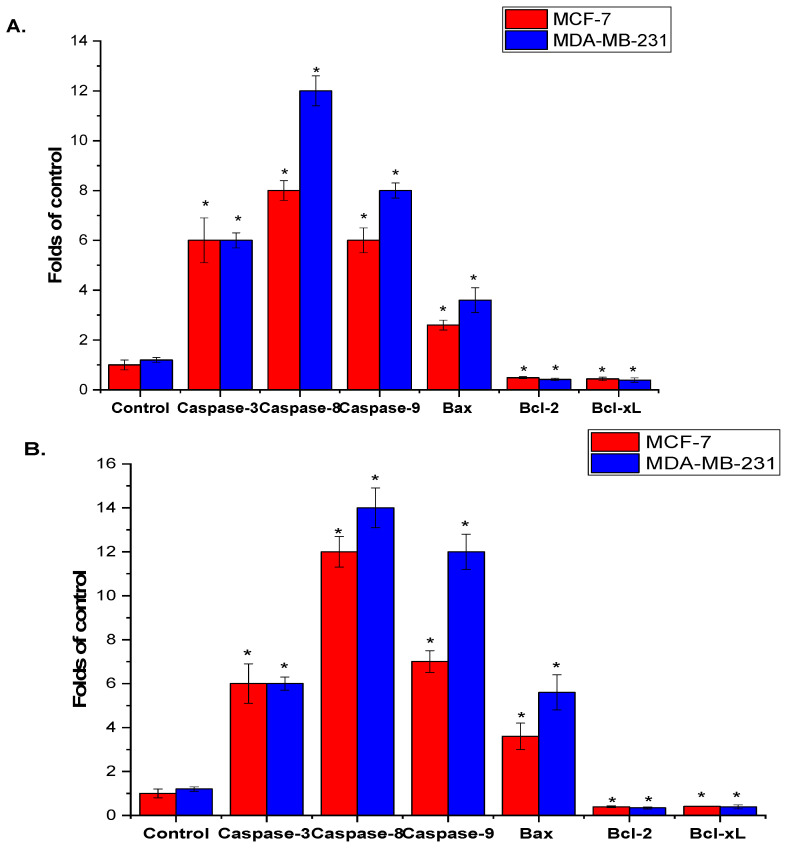
Illustration of the qPCR-based mRNA expression analysis of selected pro- and anti-apoptosis marker genes in MCF-7 and MDA-MB-231 cells treated with extracts derived from *A. visnaga* L. roots (**A**) and seeds (**B**). The results demonstrate a statistically significant difference from the control, indicated by (* = *p* < 0.05).

The effect of *A. visnaga* L. roots and seeds extract on apoptosis signaling in MCF-7 and MDA-MB-231 cells was assessed after 48 h using qPCR. As shown in Figure 4A,B, there was a significant increase in caspase-3, -8, and -9 mRNA levels in MCF-7 and MDA-MB-231 cells treated with roots and seeds extracts of *A. visnaga* L. compared to the control untreated cells. Furthermore, the extracts significantly upregulated Bax mRNA expression while significantly downregulating the anti-apoptotic genes *Bcl-2* and *Bcl-xL* in treated MCF-7 and MDA-MB-231 cells, relative to the control untreated cells (*p* < 0.05). It is noteworthy that MDA-MB-231 cells treated with *A. visnaga* L. roots and seeds extracts exhibited greater sensitivity than MCF-7 cells.

### 2.5. Antibacterial Activity of Roots and Seeds of A. visnaga L. Extracts

The antibacterial efficacy of methanol extracts derived from both roots and seeds of *A. visnaga* L. was systematically investigated against six selected bacterial strains, with results compared to Chloramphenicol (Table 3 and Table 4). Remarkably, the seeds of *A. visnaga* L exhibited superior antibacterial activity, demonstrating MIC values ranging from 3.81 ± 0.24 to 125 ± 7.63 μg/mL, outperforming the root extract (MIC, 7.81 ± 1.74 to 62.5 ± 3.53 μg/mL). Among the strains, S. *epidermidis* (MTCC 12228) and *S. aureus* were the most sensitive to seed extract with MIC (3.81 ± 0.28 and 3.81 ± 0.24 μg/mL, respectively). Weaker antibacterial effects were observed against *P. aeruginosa* (MTCC 27853) with MIC values of (62.5 ± 3.53 μg/mL) and MBC of (125 ± 5.69 μg/mL) when tested with seeds of *A. visnaga* L. extract. Interestingly, Gram-negative bacteria, including *P. aeruginosa* (MTCC 27853), *K. pneumoniae* (MTCC 13883), and *E. coli* (ATCC 25922), displayed lower sensitivity compared to Gram-positive bacteria, suggesting varied susceptibility to *A. visnaga* L. extracts.

## 3. Discussion

For centuries, pharmaceutical compounds derived from plants, including alkaloids, polyphenols, terpenoids, flavonoids, ursolic acid, paclitaxel, camptothecin, podophyllotoxin, and combretastatin, have been utilized for various purposes [41]. These natural products possess diverse properties, such as anticancer, antioxidant, anti-inflammatory, and antibacterial effects [42,43]. Despite previous studies emphasizing the cytotoxic and antibacterial effects of *A. visnaga* L. seed extracts, an unexplored avenue involves comparing the potential of root extracts as antimicrobial, antioxidant, and anticancer agents as against seed extracts. Therefore, the objective of this study was to analyze the chemical composition of methanol extracts obtained from both the roots and seeds of *A. visnaga* L. using GC/MS. Additionally, we aimed to assess the anticancer, antioxidant, and antibacterial activities of the methanol extracts derived from the roots and compare them with the seed extracts. Furthermore, we employed qPCR-based mRNA expression profiling of selected pro- and anti-apoptosis marker genes to explore the potential of apoptosis induction.

The chemical constituents of *A. visnaga* are well known and have been reported by many researchers in numerous studies throughout the years. Numerous chemical components of *A. visnaga*, notably γ-pyrones (Furanochromone Derivatives), have been the subject of previous research. The two principal ones are visnagin and khellin [44,45], together with 4-norvisnagin, khellinol, visamminol, ammiol, and khellol [46]. In addition to khellinin, khellinone, and visnaginone [10], other significant γ-pyrones are 5,7-dihydroxy-2-methyl-γ-pyrone-7-O-glucoside [12]. The most prevalent components of *A. visnaga* essential oils were also discovered to be isoamyl 2-methylbutyrate, isoamyl isobutyrate, iso-butyl-2-methylbutyrate, 2-methylbutyl 2-methylbutyrate, 2-methylbutyl isobutyrate, and isoamyl isovalerate [45,47]. Our study involved the analysis of various phytochemical compounds using GC-MS. Docosanolide (73.39%) was identified as the main photochemical component in the obtained methanol extract of roots. According to a preliminary investigation, docosanol administration had distinct effects on human melanoma and CHO-K1 cells, considerably affecting their rates of proliferation but not their viability. The composition of *A. visnaga* L. has been the subject of numerous investigations. *A. visnaga* L. was shown to contain important phytochemical substances, including (Z)-β-ocimen and oxygenated monoterpenes such D-limonene, linalool, pulegone, and lavandulyl-butyrate, in a recent study. According to [44], the major components of the essential oil derived from ripe fruits of *A. visnaga* L. were reported as nerol (29.98%), α-bisabolol (20.86%), and butylated hydroxytoluene (18.55%). The differences in chemical composition observed among *A. visnaga* L. plants may be attributed to various ecological and genetic factors. Factors such as soil type and properties, soil management practices, and environmental stress can influence the modulation of specific enzyme groups responsible for regulating biosynthetic pathways. These factors can ultimately impact the production and abundance of phytochemical compounds in the plant [10,48,49].

Significant secondary metabolites, such as quercetin, kaempferol, rhamnocitrin, and rhamnetin, are known to be present in *A. visnaga* L., predominantly in its aerial parts. Extensive research has been conducted by multiple investigators, including [12,50], to identify and study these compounds. The presence of phenolic compounds in *A. visnaga* L. contributes to its potential pharmacological activities and associated health benefits. However, the yield of these phenolic compounds in the extract solvent can be influenced by various factors. These factors include the type of solvent used, the extraction method employed, the extraction time, and temperature, as well as the composition and physical properties of the sample [51,52,53]. In the conducted study, the methanol extracts of *A. visnaga* L. roots and seeds were analyzed for their TPC. The TPC values were determined to be 270.78 ± 2.86 and 366.57 ± 2.86 ± 4.09 mg GAE dry weight of the dry extract, respectively. Additionally, the investigation included the assessment of TFC, which plays a vital role as a major plant pigment with radical scavenging activity and contributes to plant coloration. The TFC levels in *A. visnaga* L. seeds and roots were found to be 242.10 ± 3.07 and 214.46 ± 2.86 mg QE per dry weight of the dry extract, respectively. It is worth noting that the TPC and TFC values obtained in our study were found to be higher than those reported in previous studies. For instance, a recent investigation reported TPC and TFC values of 76.75 ± 2.31 mg GA per gram dry weight of the dry extract and 11.75± 0.02 mg QE per gram dry weight of the dry extract, respectively, in an ethanolic extract derived from the aerial portion of *A. visnaga* L. [54]. In contrast to the findings in this research, another recent study conducted by [55] reported different results regarding the TPC and TFC in an ethanolic extract of the aerial part of *A. visnaga* L. Specifically, their study indicated that a 70% methanolic extract exhibited the highest TPC value of 176 mg GAE per gram dry weight of the dry extract and TFC value of 22 mg QE per dry weight of the dry extract. These values differ from the TPC and TFC values obtained in our study for the roots and seeds of *A. visnaga* L. It is important to acknowledge the variability that can arise in the composition and content of phenolic compounds and flavonoids due to different extraction methods and plant sources. The analysis of the essential oil derived from *A. visnaga* L. revealed a range of TPC values from 7.26 ± 1.68 mg GA per gram. Furthermore, these ranged between 5.82 ± 0.79 mg QE per gram [19]. According to a recent study conducted by El Karkouri et al. in 2020, the crude extract of *A. visnaga* L. polyphenols obtained from Morocco’s Middle Atlas using Soxhlet extraction with 70% methanol yielded a content of 7.16 mg GAE per gram of phenolic components. This finding provides specific information on the phenolic content of the crude extract derived from *A. visnaga* L. in the mentioned geographical region, highlighting its potential phenolic composition and content [56]. The findings suggest the efficacy of alcoholic solvents for phenolic compound extraction, promoting safe human consumption [57,58]. Phenolic compounds have been investigated for their efficacy as robust hydrogen donors in the context of scavenging the DPPH radicalTop of Form [59].

Very few studies have examined the antioxidant properties of *A. visnaga* [12]. The investigation conducted in this research concerning the biological activities of extracts derived from the roots and seeds of *A. visnaga* L. revealed noteworthy antioxidant capabilities, as assessed through the scavenging of DPPH and ABTS radicals. The IC_50_ values for DPPH and ABTS scavenging activities were determined to be 193.46 ± 17.13 μg/mL and 227.19 ± 1.48 μg/mL, respectively. Moreover, the ABTS assay indicated IC_50_ values of 188.74 ± 2.69 μg/mL for the roots extract and 221.94 ± 1.32 μg/mL for the seeds extract, juxtaposed against the established reference standard, ascorbic acid (IC_50_ of 83.45 ± 1.24 μg/mL). Consistent with antecedent research, the findings presented herein align with a previous investigation wherein an ethanolic extract derived from the aerial component of *A. visnaga* L. demonstrated the utmost efficacy in scavenging DPPH radicals, registering a prominent activity level of 89.21% at a concentration of 3 mg/mL. This observation notably diverged from the outcomes associated with ethanol and acetone extracts at the corresponding concentration [55]. In a recent investigation, the essential oil exhibited a diminished capacity to attenuate the concentration of the DPPH free radicals, as evidenced by an obtained IC_50_ value of 4.13 ± 0.22 mg/mL. This potency stands in notable contrast to the reference antioxidant, butylated hydroxytoluene, which demonstrated a significantly superior IC_50_ value of 0.17 ± 0.01 mg/mL [19].

Flavonoids, renowned for their antioxidant and anticancer properties, play a role in inducing apoptosis, autophagy, and reducing cancer cell proliferation and invasiveness [60]. The current study aimed to assess the cytotoxic and apoptotic effects of *A. visnaga* L. roots and seeds extracts on MCF-7 and MDA-MB-231 cells. Earlier research highlighted the anticancer potential of flavonoids in *A. visnaga* leaf extract against human breast cancer BT-20 cells [61]. In a separate study, the antineoplastic efficacy of naphthoquinone was elucidated through its isolation from the leaves of the plant, specifically in the context of its impact on laryngeal cancer cells [62]. The anticancer impact of *A. visnaga* L. seeds extract was more prominent at higher concentrations, with an IC_50_ of 87.35 ± 2.44 and 72.49 ± 1.36 μg/mL for MCF-7 and MDA-MB-231 cells, respectively. In comparison, the root extract had a lower cytotoxic effect than the seed extract, with IC_50_ of 92.45 ± 2.14 and 75.43 ± 2.32 μg/mL against MCF-7 and MDA-MB-231 cells.

In a recent study, methanolic crude extracts from *A. marina*’s L. leaves exhibited potent anticancer activity against MDA-MB 231, with an IC_50_ of 480 µg/mL, showcasing superior effects compared to other extracts. The methanolic extract induced a time-dependent reduction in cell growth, suppressing it by 40%, 44%, and 59% after 24, 48, and 72 h, respectively [63]. Another study found that *A. visnaga* L. seed extract significantly decreased the viability of human hepatic carcinoma (HuH-7 cells) at concentrations of 1000 µg/mL and higher after 48 h [33].

Apoptosis, or programmed cell death, is intricately connected to various disorders, including cancer [64]. This fundamental biological process involves three primary pathways: the intrinsic pathway, regulated by mitochondria; the extrinsic pathway, mediated by death receptors; and endoplasmic reticulum stress-dependent signaling transduction. Caspase-8 serves as a mediator in the extrinsic pathway, activating executioner caspases -6 and -7 to initiate apoptosis. Simultaneously, caspase-9 acts as an important upstream mediator in the intrinsic route, initiating apoptosis by activating caspases -3 and -7 [65]. Caspase-3 engages in interactions with both caspase-8 and caspase-9, thereby showcasing its involvement in both exogenous and endogenous apoptotic properties [66]. Our RT-qPCR results showed that methanol extracts from *A. visnaga* L. root and seed, when applied to MCF-7 and MDA-MB-231 cells, increased the expression of caspase-3, -8, -9, and Bax mRNA levels while decreasing the expression of anti-apoptotic genes Bcl-2 and Bcl-xL. An early investigation demonstrated mitochondrial membrane potential dysfunction induced by *A. visnaga* L. extract, influencing the transcriptional overexpression of Bax and Bcl-2 [67]. In line with this, a recent study suggested that *A. visnaga* L. seed extract was found to upregulate caspase-3 mRNA expression in treated HuH-7 cells while downregulating Bcl-2 gene expression [33].

The rise of multidrug-resistant bacteria poses a significant challenge in treating infections and pathogenic diseases, emphasizing the urgent need to explore novel antimicrobial compounds for combatting these pathogens. Antimicrobial resistance (AMR) stands as a growing global public health threat, impacting individuals worldwide. It denotes an upsurge in the prevalence of hazardous microorganisms, including bacteria and fungi, that have developed resistance to one or more antimicrobial treatments, often referred to as multi-resistant organisms. Antibiotic resistance can manifest through various mechanisms, such as gene mutations, the production of antibiotic hydrolytic enzymes, or the horizontal gene transfer (HGT) of resistance-conferring genes. HGT, predominantly occurring between bacteria of the same species, is recognized as a major contributor to the current AMR crisis [68].

Despite significant efforts made over the past decade, natural plant-derived compounds persist as crucial and effective sources of antibacterial and anticancer agents [4]. Globally, approximately 200 plant species are recognized as medicinal plants, and they play a substantial role in the production of modern medications. These plants contribute to the synthesis of an impressive 25% to 50% of the medicines available today [9]. This study explored the antibacterial potential of methanol extracts from both roots and seeds of *A. visnaga* L., revealing potent antibacterial activity against tested bacteria. Notably, *A. visnaga* L. seeds exhibited remarkable antibacterial activity, with a MIC range from 3.81 ± 0.24 to 125 ± 7.63 μg/mL, compared to the root extract (MIC, 7.81 ± 1.74 to 62.5 ± 3.53 μg/mL). *S. epidermidis* (MTCC 12228) and *S. aureus* were notably more susceptible to the seeds with MIC (3.81 ± 0.28 and 3.81 ± 0.24 μg/mL, respectively) and roots extracts of *A. visnaga* L. with MIC (7.81 ± 0.54 and 7.81 ± 1.74 μg/mL, respectively), compared to other bacteria. Conversely, the lowest activity was observed against *P. aeruginosa* (MTCC 27853), *K. pneumoniae* (MTCC 13883), and *E. coli* (ATCC 25922), suggesting Gram-negative bacteria’s greater resistance to *A. visnaga* L. for both extracts, a phenomenon linked to the structural characteristics of the Gram-negative cell wall [69]. Numerous studies have been conducted to explore the antibacterial properties of *A. visnaga* L. extracts. In one particular investigation, the ethanolic extract derived from the fruit of *A. visnaga* L. demonstrated remarkable efficacy against the Gram-positive bacterium *Enterococcus faecalis*, with a MIC value of 5 mg/mL. Additionally, this extract exhibited antibacterial activity against Gram-negative bacteria, including *E. coli* and *K. pneumoniae*, with a MIC value of 12.5 mg/mL [27]. Earlier research also indicated antibacterial potential in the aerial sections of *A. visnaga* L. extracts, particularly the methanolic extract at 10 mg/mL, which demonstrated efficiency against B. cereus strains for swarming motility and biofilm formation [55]. Numerous researchers have conducted investigations on the antibacterial activities of *A. visnaga* L. essential oils, and their effectiveness against various pathogens has been demonstrated. These studies have shown that *A. visnaga* L. essential oils exhibit notable efficacy against pathogens such as *E. coli*, *P. aeruginosa,* and *K. pneumoniae* strains [20,47,70].

In the field of in vivo research, an early study assessed the toxicity of plant extracts, which is a critical step in guaranteeing their safety for ingestion. This entails finding safe levels that do not injure cells, tissues, or organisms [71]. In a recent study involving female Wistar rats, the administration of a 1 g/kg dosage of hydroalcoholic *A. visnaga* L. extract orally for 90 days did not show any significant impact on the liver, kidneys, or spleen. Additionally, the hematological examination revealed no noteworthy changes except for an increase in white blood cell count [72]. Furthermore, a previous investigation on Albino rats was also demonstrated that *A. visnaga* L. seed ethanolic extract, administered at doses of 150, 300, and 600 mg/kg bw/d for 14 days, did not induce organ toxicity (liver, kidney, brain, spleen, heart, testis, and ovary) or mortality [73].

One limitation of this study is the absence of in vivo experiments, as they were not the primary focus of our research. Further investigations, encompassing both in vitro and in vivo trials, are essential to comprehensively explore the putative bioactivity of *A. visnaga* L. against infectious diseases.

## 4. Materials and Methods

### 4.1. A. visnaga L. Root and Seeds Extract Preparation

The dried plant material, comprising roots and seeds of *A. visnaga* L., was procured from a supermarket located in Riyadh, Saudi Arabia. Using a mortar and pestle, a regular blender, and an electric sieve system, the plant material was meticulously crushed into a fine powder. The preparation followed a previously established protocol [74]. In summary, approximately 300 g of *A. visnaga* L. seed and root powder were separately placed in glass jars and soaked in 1000 mL of 95% methanol. These mixtures were then subjected to agitation on a rotary shaker for 72 h. After the designated time, the mixtures were filtered through a Whatman filter paper, and the filtrates were dried at 80 °C until complete evaporation of the methanol, resulting in the crude extract of *A. visnaga*. The extracts were subsequently weighed and dissolved in methanol to achieve the desired concentration. The extracts were then diluted with dimethylsulfoxide (DMSO) (Sigma-Aldrich, Ayrshire, UK) at a concentration of 0.1% and used as a negative control. Finally, the prepared extracts were stored in sealed vials, protected from light, at a temperature of 4 °C, until further utilization.

### 4.2. Determination of Phytochemicals from the Methanol Roots and Seeds Extracts of A. visnaga L.

The methanol extracts of *A. visnaga* L. roots and seeds were subjected to analysis using a Chromatography-mass spectrometry (GC–MS) system manufactured by Agilent Technologies Inc. Stevens Creek Blvd, Santa Clara, CA, USA. The GC–MS system was equipped with an Agilent 5977A MSD system. The volatile compounds present in the methanol extract were purified using a capillary column with dimensions of 30 m × 0.25 mm and a 0.25 μm film thickness. Helium gas was employed as the carrier gas, with a flow rate of 0.5 mL/min. During the analysis, the injector temperature was maintained at 250 °C. The oven temperature was programmed as follows: initially, it was held at 70 °C for 3 min, then increased by 3 °C/min to reach 100 °C, and held for 3 min. Subsequently, the temperature was further increased to 120 °C at a rate of 10 °C/min and maintained for 3 min. Finally, the temperature was raised to 220 °C at a rate of 10 °C/min. The mass spectrum settings included an electron impact (EI) source, with the ionization temperature set at 230 °C and the electron energy at 70 eV. The quadrupole temperature was maintained at 150 °C, and the interface temperature was set to 280 °C. The quantity scanning range for the mass spectrum analysis ranged from 20 amu to 500 amu.

### 4.3. Antioxidant Activity

#### 4.3.1. Analysis of TPC

The TPC of *A. visnaga* L. roots and seeds was determined using the Folin-Ciocalteu method, as described in a previous study conducted by [75]. In brief, approximately 0.05 mL of the *A. visnaga* L. root and seed extracts were separately mixed with 0.25 mL of Folin-Ciocalteau reagent (0.5 N), and the mixture was incubated for 10 min. Next, 2.5 mL of 0.5 M sodium carbonate solution was added, followed by an additional 30-min incubation. The optical density (OD) of the samples was measured at 765 nm against a reagent blank with a VR-2000.0 spectrophotometer (JP Selecta, Barcelona, Spain). Gallic acid was used as a standard at concentrations ranging from 10 to 100 mg/mL. The TPC was calculated as mg GAE per gram of extract at various concentrations, as mentioned in that study.

#### 4.3.2. Analysis of TFC

The method described by Ordonez et al. was employed to assess the TFC in the extracts of *A. visnaga* L. roots and seeds [76]. In this method, approximately 0.1 mL of the *A. visnaga* L. root and seed extracts were separately incubated with 2.0 mL of a 2% AlCl3 solution for 30 min. Following incubation, the absorbance of the extract was measured using an ELX-808 microplate reader (BioTek Laboratories, LL, Shoreline, WA, USA) at a wavelength of 420 nm. A standard curve was generated using various concentrations (10–100 mg/mL) of quercetin as the reference standard. The TFC was determined by comparing the absorbance of the extracts to the standard quercetin curve. The results were expressed as mg QE/g per gram of extract.

#### 4.3.3. DPPH Scavenging Assay

The antioxidative activity of *A. visnaga* L. roots and seeds was assessed through the utilization of the DPPH (1,1-diphenyl-2-picrylhydrazyl) scavenging assay, as outlined in a study conducted by [77]. The extracts of *A. visnaga* L. roots and seeds were prepared at four different concentrations (100, 200, 400, and 800 μg/mL). For each concentration, approximately 0.5 mL of the extract was mixed with 0.375 mL of methanol and a DPPH solution (2 mL, 0.08 mM). The reaction mixture was then placed in a dark environment and incubated for 30 min. After incubation, the OD of the mixture was measured at 517 nm using a spectrophotometer. Ascorbic acid was used as a positive control at known concentrations (µg/mL). The results were reported as IC_50_ values, which were calculated using Graph Pad Prism software (version 5.0, La Jolla, CA, USA), and the percentage of DPPH scavenging activity was determined.

#### 4.3.4. ABTS Assay

The ABTS assay was performed by following the experimental procedure outlined by [78]. The assay was carried out by first mixing the ABTS solution (192 mg/50 mL) with the K2S2O8 solution (140 mM). Consequently, the reaction mixture was placed in the dark at room temperature for about 12 h. In addition, the ABTS solution was mixed with methanol to achieve an OD of 0.70 ± 0.02 at 734 nm. Furthermore, fifty microliters of each concentration of the extract (both roots and seeds) were thoroughly mixed with 3 mL of diluted ABTS. Moreover, the mixture was incubated for 6 min in dark conditions. Subsequently, the OD was measured at 734 nm using a VR-2000.0 spectrophotometer (JP Selecta, Barcelona, Spain). It is worth mentioning that the positive control utilized in this study was ascorbic acid (µg/mL). Lastly, the results were represented as percentages of DPPH and IC50 values.

### 4.4. Cell Culture and Cytotoxicity Assays

The two types of breast cancer cells, namely, MCF-7 (ATCC HTB-22) and MDA-MB-231 (ATCC HTB-26), were cultured in DMEM (Qiagen, Germantown, MD, USA). Moreover, the culture media were supplemented with 1% penicillin/streptomycin and 10% fetal bovine serum (FBS). Additionally, the cells were maintained at a temperature of 37 °C in a humidified atmosphere with 5% CO_2_. Furthermore, the media were changed every 2–3 days to ensure optimal cell growth, as outlined in a study conducted by [79]. To assess the cytotoxicity of *A. visnaga* L. root and seed extracts, the MTT assay was conducted on MCF-7 and MDA-MB-231 cells. This method relies on mitochondrial dehydrogenases in living cells to convert MTT into formazan crystals [80,81]. First, the cells were seeded in 96-well microtiter plates at a density of 1 × 10^4^ cells per well. Subsequently, after 24 h of incubation at 37 °C in a 5% CO_2_ atmosphere, fresh media were added to the wells. In addition, a range of different concentrations of *A. visnaga* L. root and seed extracts (ranging from 50 μg/mL to 400 μg/mL) were carefully added to the wells. The plates were then incubated at 37 °C for 24 h under the same aforementioned conditions. After the 24-h incubation period, approximately 10 µL of MTT reagent (5 mg/mL) was meticulously added to each well. Moreover, a further incubation of 30 min was conducted. Consequently, 100 μL of DMSO was added to the wells and incubated for an additional 10 min. The absorbance of the samples was measured using a microtiter plate reader at a wavelength of 590 nm using an ELX-808 microplate reader (BioTek Laboratories, LL, Shoreline, WA, USA), with positive and negative controls used for comparison. The cell viability (%) was then calculated. Notably, the experiment was performed in triplicate to ensure reliable results. The mean value was considered for data analysis, and the IC_50_ value was subsequently determined using the GraphPad Prism software.

### 4.5. Apoptosis Coding Genes Assay

The evaluation of apoptosis coding genes was carried out as originally described by [34], with minor modifications. Precisely 2 × 10^5^ MCF-7 and MDA-MB-231 cells were seeded into six-well plates containing 3 mL of culture medium. The culture medium was then mixed with roots or seeds extracts of *A. visnaga L* (100 μg/mL), respectively. After a 48-h incubation at 37 °C, the wells were washed with phosphate-buffered saline (PBS) and trypsinized using 0.25% trypsin prepared in 0.53 mM EDTA. The cells were centrifuged at 500× *g* for 5 min at 4 °C, and the upper liquid phase was discarded. The resulting pellet was used for rRT-PCR analysis to assess the expressions of apoptosis-coding genes.

To extract cellular RNA from the cancer cells, an RNeasy Micro Kit (Qiagen, Hilden, Germany) was used following the manufacturer’s instructions. The extracted RNA was then subjected to qPCR using the one-step RT^2^ SYBR^®^ Green/ROX™ qPCR Master Mix. For each sample, a master mix was prepared by combining the following components: Go Taq Qpcr master mix (2×) (10µL), one-step RT mix (0.4 µL), CXL (0.33 µL), F primer (0.4 µL), R primer (0.4 µL), RNase-free water (8.5 µL), and RNA template (4 µL). The mixture was thoroughly mixed, centrifuged, and transferred to the RT^2^ PCR Array Loading Reservoir. The RT^2^ Profiler PCR Array was tightly sealed with optical thin Wall Cap Strips and placed in a pre-programmed real-time instrument (7500 Applied Biosystems).

The rRT-PCR reactions were carried out in a 7500 Fast real-time PCR (7500 Fast; Applied Biosystems, Foster City, CA, USA) using the following cycling conditions: reverse transcription at 37 °C for 15 min, and initial PCR denaturation at 95 °C for 10 min. Each PCR cycle consisted of denaturation at 95 °C for 10 s, annealing at 60 °C for 30 s, and extension at 72 °C for 30 s. A total of forty cycles were performed. Melting curve analysis was performed to confirm the specificity of amplification and to ensure there were no primer dimers present. The results were analyzed using the 2^−ΔΔCq^ method, as described by [82]. Furthermore, Delta Cq (ΔCq) values were obtained for the different genes and, to normalize the data, these values were based on the expression of GAPDH amplified from the same samples. The fold change in expression was then calculated by comparing it to the expression of the untreated control cells. The specific oligo sequences used for the PCR amplification can be found in Table 5.

### 4.6. Antibacterial Activity

#### 4.6.1. Microbial Strain

The antibacterial activity of the root and seed methanol extracts of *A. visnaga* was evaluated against several bacterial pathogens that included both Gram-positive and Gram-negative strains. In addition, the selected Gram-positive strains, such as *Bacillus subtilis* (MTCC-10400), Staphylococcus aureus (MTCC-29213), and *Staphylococcus epidermidis* (MTCC-12228), were included in this study. Additionally, three Gram-negative strains, namely *Klebsiella pneumoniae* (MTCC-13883), *Escherichia coli* (ATCC-25922), and *Pseudomonas aeruginosa* (MTCC-27853), were utilized. It is important to note that all of these bacterial strains were obtained from the King Khalid University Hospital in Riyadh, Saudi Arabia.

#### 4.6.2. Disc Diffusion Method

The agar disc diffusion method, widely recognized as the gold standard, was employed in this study, following previously established procedures [87] with minor adaptations. Specifically, we utilized methanol extracts derived from *A. visnaga* L. root and seed for our analysis. Our investigation into the antimicrobial activity was conducted on nutrient agar. To cultivate the tested bacteria, they were initially incubated in nutrient agar for 24 h at 37 °C. Subsequently, a bacterial inoculum of 1 × 10^6^ CFU/mL saline was incubated on nutrient agar for another 24 h. For the application of the bacterial inoculum, approximately 0.1 mL was evenly spread on nutrient agar plates using an “L” rod. To assess the impact of the methanol extract of *A. visnaga* L. root and seed, 20 µg of each extract was placed on separate filter paper discs (6 mm in diameter) and then incubated for 24 h. It’s important to note that Chloramphenicol (20 μg/mL) served as our positive control, while a 0.1% DMSO solution in nutrient broth was used as the negative control. The resulting zones of inhibition surrounding each well-containing methanol extract of *A. visnaga* L. root and seed were measured in diameter following an overnight incubation [88].

#### 4.6.3. Determination of MIC and MBC

The antibacterial activity of methanol extracts obtained from the roots and seeds of *A. visnaga* L. was investigated concerning both Gram-positive and Gram-negative bacteria. The evaluation was carried out using the MIC and MBC methods of broth dilution, as originally described by [89]. However, minor modifications were implemented in the assay procedure for this study. The assay was carried out employing microtiter plates. Firstly, approximately 300 µL of nutrient broth medium was added to each well. Subsequently, a series of dilutions of the root and seed extracts (ranging from 1.56 to 800 μg/mL) were separately added to different plates. Following this, 20 µL of inoculum containing 1 × 10^5^ CFU/mL was added to all the wells. The microtiter plates were then incubated at 37 °C for 18 h. The presence or absence of bacterial growth was determined by visually observing changes in turbidity. Hence, meticulous examination of all the wells in the microtiter plate was conducted to discern any discernible alterations in turbidity, which would indicate bacterial growth. It is noteworthy that Chloramphenicol (20 μg/mL) and 0.1% DMSO served as the positive and negative controls, respectively. MBC determinations were confirmed by spreading the culture from wells that showed no growth onto nutrient agar plates, following the methodology described by [90].

### 4.7. Statistical Analysis

To analyze the data, a One-Way ANOVA was conducted, and a significant level was observed. It is important to note that all experiments were performed in triplicates, and the mean standard deviation (SD) was utilized for the analysis. To determine statistical significance, a threshold of “*p*” value > 0.05 was adopted.

## 5. Conclusions

In this study, the primary objective was to evaluate the antioxidant, anticancer, and antibacterial properties of a methanol extract obtained from the roots and seeds of *A. visnaga* L. To this end, the phenolic compounds and flavonoids present in *A. visnaga* L. were analyzed, suggesting its potential as an antioxidant agent. Comparing the roots and seeds of *A. visnaga* L. to the positive control, we observed significant antioxidant, cytotoxic, and antibacterial activities in both samples. The methanolic extracts from both roots and seeds exhibited heightened antioxidant activity, with the seeds extract displaying superior antioxidant, anticancer, and antibacterial effects compared to the root extract. These findings collectively suggest that *A. visnaga* extracts harbor antioxidant, cytotoxic, and antibacterial properties, indicating their potential clinical use as therapeutic agents for reducing disease risk.

## Figures and Tables

**Table 1:** GC-MS identified volatile components from the methanol roots extract of *A. visnaga* L.

Peak	Compound Name	Retention Time (min)	Peak Area %	Area
1	1-Butoxy-2-Propanol Acetate	5.04	5.36	14,009
2	5-Methyl-2-Heptanamine	6.97	8.22	21,484
3	(-)-Curcuhydroquinone	9.39	9.03	31,466
4	Di-Isodecyl Phthalate	9.76	3.66	9559
5	2,5-Dimethyl-5-Nitrohexanal	12.56	10.70	27,988
6	3-Methyl-2h-Cyclohepta[B]Furan	14.98	2.85	59,272
7	Angecin	19.24	10.75	223,303
8	9-Octadecenoic Acid	20.52	8.11	168,542
9	6-Methoxy-2-Oxo-(2h)-Furo[2,3-H]-1-Benzopyran	21.60	1.54	32,007
10	Docosanolide	22.32	73.39	1,524,375

**Table 2:** GC-MS identified volatile components from the methanol seeds extract of *A. visnaga* L.

Peak	Compound Name	Retention Time (min)	Peak Area %	Area
1	Camphor	9.59	0.68	151,747
2	4-Methyl-1-Methyl 3-Cyclohexen-1-Ol	10.12	0.75	167,952
3	3-Cyclohexene-1-Methanol	10.35	4.04	908,773
4	4-Phenyl2-Butanone	11.00	3.34	750,682
5	Isocaryophyllen	13.76	0.51	114,170
6	Trans-*α*-Bergamotene	13.87	1.14	256,370
7	3-Methyl-2h-Cyclohepta[B]Furan	14.98	2.85	59,272
8	2-Benzoyl-3-Methyl-2,3-Diaza	18.17	54.60	450,1224
9	1-Methyl-5-Phenyltetrazole	18.34	1.92	158,385
10	2-Methoxy-4-Propyl-Phenol	18.54	24.96	2,057,664
11	Neophytadiene	19.09	0.510	42,337
12	Angecin	19.24	5.66	466,675
13	6-Octadecenoic Acid	22.22	10.68	880,313

**Table 3:** The inhibitory zone, MIC, and MBC of roots of *A. visnaga* L. extracts.

Bacterium/Dilution	Positive Control	500 μg/mL	250 μg/mL	125 μg/mL	62.5 μg/mL	MIC (μg/mL)	MBC (μg/mL)
*S. aureus* (MTCC 29213)	25 ± 1.24	21 ± 1.62	18 ± 0.64	14 ± 1.38	12 ± 1.36	7.81 ± 1.74	15.62 ± 3.84
*S. epidermidis* (MTCC 12228)	26 ± 1.14	22 ± 2.34	19 ± 2.36	14 ± 0.98	12 ± 1.39	7.81 ± 0.54	15.62 ± 1.83
*B. subtilis* (MTCC 10400)	24 ± 0.42	21 ± 3.93	19 ± 1.44	13 ± 1.65	11 ± 1.69	15.62 ± 1.43	31.25 ± 1.23
*E. coli* (ATCC 25922)	25 ± 2.15	20 ± 1.64	15 ± 1.66	12 ± 2.37	6 ± 0.39	31.25 ± 1.23	62.5 ± 3.76
*K. pneumoniae* (MTCC 13883)	22 ± 1.35	17 ± 132	13 ± 1.25	8 ± 1.45	6 ± 0.93	31.25 ± 1.45	62.5 ± 3.53
*P. aeruginosa* (MTCC 27853)	28 ± 2.27	19 ± 0.64	14 ± 0.44	11 ± 1.27	5 ± 0.83	62.5 ± 3.53	125 ± 5.69

**Table 4:** The inhibitory zone, MIC, and MBC of seeds of *A. visnaga* L. extracts against selected bacteria strains.

Bacterium/Dilution	Positive Control	500 μg/mL	250 μg/mL	125 μg/mL	62.5 μg/mL	MIC (μg/mL)	MBC (μg/mL)
*S. aureus* (MTCC 29213)	25 ± 1.24	18 ± 2.65	16 ± 1.33	12 ± 2.36	8 ± 0.69	3.81 ± 0.24	7.81 ± 2.43
*S. epidermidis* (MTCC 12228)	26 ± 1.14	23 ± 0.98	18 ± 1.36	12 ± 1.93	8 ± 0.76	3.81 ± 0.28	7.81 ± 1.34
*B. subtilis* (MTCC 10400)	24 ± 0.42	20 ± 1.35	12 ± 1.95	10 ± 0.64	9 ± 0.36	15.63 ± 1.63	31.25 ± 2.65
*E. coli* (ATCC 25922)	25 ± 2.15	18 ± 0.59	14 ± 1.25	10 ± 1.44	8 ± 0.35	50 ± 5.73	100 ± 3.57
*K. pneumoniae* (MTCC 13883)	22 ± 1.35	18 ± 0.92	11 ± 0.84	9 ± 0.39	5 ± 0.23	125 ± 7.63	250 ± 5.66
*P. aeruginosa* (MTCC 27853)	28 ± 2.27	17 ± 0.85	12 ± 0.37	9 ± 0.33	6 ± 0.25	31.25 ± 5.27	62.5 ± 4.65

**Table 5:** The primer sequences of various genes involved in apoptosis and anti-apoptotic genes.

Gene Name	Primers Sequence	Reference
Caspase-3	F: 5′-GCTGGATGCCGTCTAGAGTC-3′	[83]
	R: 5′-ATGTGTGGATGATGCTGCCA-3′	
Caspase-8	F: 5′-AGAAGAGGGTCATCCTGGGAGA-3′	[84]
	R: 5′-TCAGGACTTCCTTCAAGGCTGC-3′	
Caspase-9	F: 5′-ATTGCACAGCACGTTCACAC-3′	[83]
	R: 5′-TATCCCATCCCAGGAAGGCA-3′	
Bax	F: 5′-GAGCTAGGGTCAGAGGGTCA-3′	[83]
	R: 5′-CCCCGATTCATCTACCCTGC-3′	
Bcl-2	F: 5′-ACCTACCCAGCCTCCGTTAT-3′	[83]
	R: 5′-GAACTGGGGGAGGATTGTGG-3′	
Bcl-XL	F: 5′-CAGAGCTTTGAACAGGTAG-3′	[85]
	R: 5′-GCTCTCGGGTGCTGTATTG-3′	
	R: 5′-GGGCGGATTAGGGCTTCC-3′	
GAPDH	F: 5′-CGGAGTCAACGGATTTGGTC-3′	[86]
	R: 5′-AGCCTTCTCCATGGTCGTGA-3′	

## Data Availability

Data is contained within the article.
